# Running-specific prosthesis model, stiffness and height affect biomechanics and asymmetry of athletes with unilateral leg amputations across speeds

**DOI:** 10.1098/rsos.211691

**Published:** 2022-06-01

**Authors:** Joshua R. Tacca, Owen N. Beck, Paolo Taboga, Alena M. Grabowski

**Affiliations:** ^1^ Paul M. Rady Department of Mechanical Engineering, University of Colorado, Boulder, CO, USA; ^2^ Department of Integrative Physiology, University of Colorado, Boulder, CO, USA; ^3^ George W. Woodruff School of Mechanical Engineering, Georgia Institute of Technology, Atlanta, GA, USA; ^4^ School of Biological Sciences, Georgia Institute of Technology, Atlanta, GA, USA; ^5^ Department of Kinesiology, Sacramento State University, Sacramento, CA, USA; ^6^ Department of Veterans Affairs, Eastern Colorado Healthcare System, Denver, CO, USA

**Keywords:** prostheses, sprinting, amputee, symmetry, transtibial

## Abstract

Athletes with transtibial amputation (TTA) use running-specific prostheses (RSPs) to run. RSP configuration likely affects the biomechanics of such athletes across speeds. We determined how the use of three RSP models (Catapult, Sprinter and Xtend) with three stiffness categories (recommended, ±1), and three heights (recommended, ±2 cm) affected contact length (*L_c_*), stance average vertical ground reaction force (*F*_avg_), step frequency (*f*_step_) and asymmetry between legs for 10 athletes with unilateral TTA at 3–7 m s^−1^. The use of the Xtend versus Catapult RSP decreased *L_c_* (*p* = 2.69 × 10^−7^) and *F*_avg_ asymmetry (*p* = 0.032); the effect on *L_c_* asymmetry diminished with faster speeds (*p* = 0.0020). The use of the Sprinter versus Catapult RSP decreased *F*_avg_ asymmetry (*p* = 7.00 × 10^−5^); this effect was independent of speed (*p* = 0.90). The use of a stiffer RSP decreased *L_c_* asymmetry (*p* ≤ 0.00033); this effect was independent of speed (*p* ≥ 0.071). The use of a shorter RSP decreased *L_c_* (*p* = 5.86 × 10^−6^), *F*_avg_ (*p* = 8.58 × 10^−6^) and *f*_step_ asymmetry (*p* = 0.0011); each effect was independent of speed (*p* ≥ 0.15). To minimize asymmetry, athletes with unilateral TTA should use an Xtend or Sprinter RSP with 2 cm shorter than recommended height and stiffness based on intended speed.

## 1. Introduction

**Figure 1.**
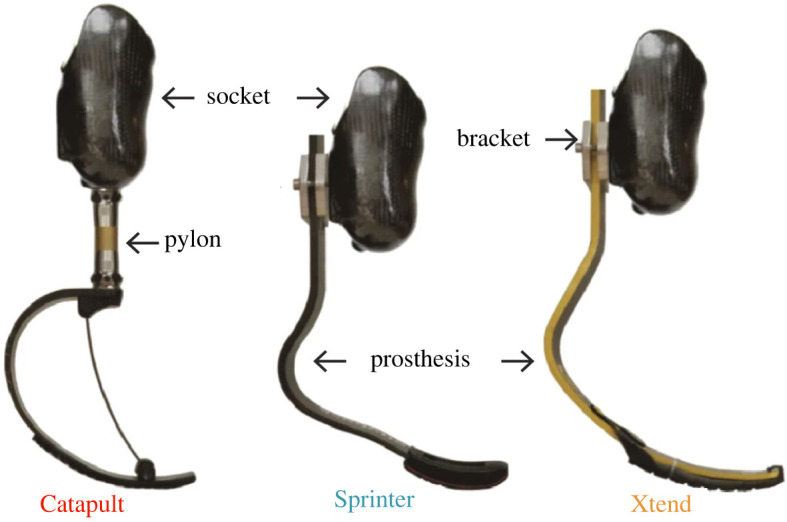
RSP models used in this study. (*a*) Freedom Innovations Catapult FX6 C-shaped RSP, (*b*) Ottobock 1E90 Sprinter J-shaped RSP and (*c*) Össur Cheetah Xtend J-shaped RSP. The height of the C-shaped RSP was adjusted by changing the pylon length. The heights of the J-shaped RSPs were adjusted using a custom aluminium bracket.

Athletes with a transtibial amputation (TTA) use a running-specific prosthesis (RSP) to run. An RSP is composed of carbon fibre and attached in-series with a rigid socket that surrounds the residual limb. Previous studies show that the configuration of an RSP (e.g. model) can affect performance, specifically the metabolic cost and maximum speed, of an athlete with TTA [1–3]. The model of an RSP is generally shaped like a ‘C’ or ‘J’ (figure 1). C-shaped RSPs are positioned beneath the socket and attach via a metal pylon and J-shaped RSPs are positioned behind and attach directly to the socket. Manufacturers typically recommend C-shaped RSPs to athletes who wish to run longer distances and J-shaped RSPs to athletes who wish to sprint [4]. In addition, prosthetists prescribe an RSP with a stiffness category based on the manufacturer's recommendation for an athlete's body mass and activity level, as well as through visual inspection of symmetric ground contact time between legs [4–7]. Each manufacturer-established RSP stiffness category has a corresponding stiffness value in kN m^−1^ [8], but the prescribed stiffness value varies between RSP models [8]. Furthermore, the height of an RSP can be adjusted by changing the length of the pylon for a C-shaped RSP or the attachment position of a J-shaped RSP. For athletes with unilateral TTA, the height of an RSP is typically set by a prosthetist based on visual inspection of the hips being level during running and an athlete's personal preference [5]. Usually, the unloaded affected leg length is set 2–8 cm longer than the standing unaffected leg length [9].

The biomechanical variables of each leg interact to influence running speed, which equals the product of stride frequency and stride length. A stride is comprised of two steps, where a step is the ground contact phase and subsequent aerial phase of one leg [9]. Step frequency (*f*_step_) is the reciprocal of step time. Step length equals the distance the centre of mass moves forward during ground contact (*L_c_*) and during the aerial phase. Aerial time and therefore the distance travelled forward during the aerial phase is lengthened by increasing stance average vertical ground reaction force (GRF) normalized to bodyweight (*F*_avg_), and thus step length can be derived as the product of *L_c_* and *F*_avg_ [3]. Running speed is therefore the product of *f*_step_, *L_c_* and *F*_avg_ [3]. These variables differ between the legs of athletes with unilateral TTA. For example, Grabowski *et al*. [9] found that athletes with unilateral TTA exhibited 9% lower *F*_avg_ in their affected leg than their unaffected leg across a range of speeds (3 m s^−1^ to maximum speed). Ultimately, athletes with unilateral TTA using an RSP often exhibit biomechanical asymmetries between their legs in spatio-temporal variables [1,2,10,11], GRFs [1,2,9,11–14], impulses [12,15], joint moments [16,17] and leg stiffness [14,18] during running. Biomechanical asymmetries have been associated with secondary injuries such as a hamstring strain and osteoarthritis [19,20]. Thus, determining the RSP model, stiffness and height configuration that decreases biomechanical asymmetry at a given running speed could alleviate or decrease the risk of injury in athletes with a TTA.

Previous studies have examined the effects of different RSP model, stiffness category and height configurations on metabolic cost and maximum speed of athletes with unilateral TTA to determine how RSP configuration affects performance as well as the underlying biomechanics [1,2]. Overall, the use of J-shaped RSPs resulted in lower metabolic cost at 2.5 and 3 m s^−1^ and faster maximum speed compared with C-shaped RSPs, but RSP stiffness category and height did not affect performance [1,2]. At 2.5 and 3 m s^−1^ use of the J-shaped Ottobock 1E90 Sprinter (Duderstadt, Germany, figure 1) compared with the C-shaped Freedom Innovations Catapult FX6 (Irvine, CA, USA, figure 1) RSP did not change overall *L_c_* (average *L_c_* of the unaffected and affected legs), but increased overall *F*_avg_ [1]. Similarly, at maximum speed use of the Sprinter RSP did not change overall *L_c_* but increased overall *F*_avg_ and decreased overall *f*_step_ compared with the use of the Catapult RSP [2]. However, at maximum speed use of the J-shaped Össur Cheetah Xtend (Reykjavik, Iceland, figure 1), RSP decreased overall *L_c_*, increased overall *F*_avg_ and decreased overall *f*_step_ compared with the use of the Catapult RSP. Thus, RSP model affects overall biomechanics at 2.5 and 3 m s^−1^ and maximum speed. The use of stiffer RSP categories decreased overall *L_c_* and increased overall *F*_avg_ at 2.5 and 3 m s^−1^ [1], but the effect of RSP stiffness categories on these biomechanical variables at maximum speed are unknown. The use of a 2 cm taller versus shorter than recommended RSP height did not affect overall *L_c_* and *F*_avg_, but resulted in more asymmetric peak vertical GRF when running at 2.5 and 3.0 m s^−1^ [1]. Thus, RSP stiffness and height also influence overall biomechanics and asymmetry at 2.5 and 3 m s^−1^ and may influence overall biomechanics and asymmetry at maximum speed. However, the effects of RSP model, stiffness and height configurations on individual leg biomechanics and biomechanical asymmetry across a range of running speeds are unknown. There is likely a relationship between RSP configuration and the biomechanics that influence speed over a range of running speeds for athletes with unilateral TTA. Thus, we determined the effects of different RSP model, stiffness and height on the affected leg biomechanics and biomechanical asymmetry of athletes with unilateral TTA across a range of speeds to establish the RSP configuration that minimizes biomechanical asymmetry. By measuring both affected leg biomechanics and biomechanical asymmetry, we characterized the effects of different RSP configurations on each leg. Our results will inform RSP prescription and design and may reduce injury risk for athletes with unilateral TTA running at a range of speeds.

We quantified the effects of using 15 different RSP model, stiffness and height configurations on the affected leg biomechanics and biomechanical asymmetry of athletes with a TTA across a range of speeds. Regarding RSP model and based on previous studies, we hypothesized that (1a) the use of the Xtend versus Sprinter and Catapult RSPs would elicit shorter affected leg *L_c_*, the use of the Sprinter and Xtend versus Catapult RSPs would increase affected leg *F*_avg_ and decrease affected leg *f*_step_, and these relationships would be independent of speed. (1b) Further, we hypothesized that the use of the Sprinter and Xtend versus Catapult RSPs would not affect *L_c_* asymmetry, would decrease *F*_avg_ asymmetry and would not affect *f*_step_ asymmetry, and these relationships would be independent of speed. Regarding RSP stiffness and based on previous studies, we hypothesized that (2a) the use of a stiffer versus less stiff RSP would decrease *L_c_*, increase *F*_avg_ and increase *f*_step_ in the affected leg, and these effects would be mitigated with faster speed. (2b) Further, we hypothesized that use of a stiffer versus less stiff RSP would decrease *L_c_*, *F*_avg_ and *f*_step_ asymmetry, and this effect would be mitigated with faster speed. Regarding RSP height and based on previous studies, we hypothesized that (3a) the use of a taller versus shorter RSP would not affect *L_c_*, *F*_avg_ or *f*_step_ of the affected leg, and these effects would be independent of speed. (3b) Further, we hypothesized that the use of a taller versus shorter RSP would increase *L_c_*, *F*_avg_ and *f*_step_ asymmetry, and this effect would be independent of speed.

## 2. Participants and methods

### 2.1. Participants

Ten healthy subjects (seven males and three females) with a TTA participated (table 1). Each subject had at least 1 year of experience using an RSP and gave informed written consent to the protocol that was approved by the Colorado Multiple Institutional Review Board and the United States Army Medical Research and Material Command Office of Research Protection, Human Research Protection Office (COMIRB #13-0559). Subjects reported no additional musculoskeletal, cardiovascular, pulmonary or neurological disease, disorder or injury beyond a TTA.

**Table 1.** Subject characteristics: sex, age, mass, standing unaffected leg (UL) length and unloaded affected leg (AL) length for the recommended RSP height for each model (Freedom Innovations Catapult FX6, Ottobock 1E90 Sprinter, and Össur Cheetah Xtend).

subjects	sex	age (years)	mass (kg)	UL length (m)	AL length with Catapult (m)	AL length with Sprinter (m)	AL length with Xtend (m)
1	male	23	82.59	1.00	1.04	1.05	1.04
2	male	25	87.87	0.88	0.94	0.92	0.96
3	female	29	75.62	0.94	1.01	0.96	1.01
4	male	31	74.38	1.01	1.04	1.03	1.03
5	male	22	71.69	0.90	0.94	0.92	0.93
6	male	33	92.66	0.99	1.05	1.05	1.05
7	male	34	66.69	0.96	1.04	1.00	1.01
8	male	37	90.73	0.95	1.02	1.01	1.00
9	female	29	58.25	0.81	0.86	0.88	0.87
10	female	21	59.51	0.84	0.88	0.88	0.88
average		28.40	76.00	0.93	0.98	0.97	0.98
s.d.		5.48	12.36	0.07	0.07	0.07	0.07

### 2.2. Protocol

This study provides further analysis of data collected during a previous study and, therefore, has the same protocol [2]. On the first day of the protocol, each subject completed an RSP alignment and accommodation session. First, we measured each subject's height, weight and leg lengths (unloaded affected leg length from the greater trochanter to the bottom of the RSP's rubber sole and standing unaffected leg length from the greater trochanter to the floor). Then, a certified prosthetist aligned subjects to three different RSP models (Össur Cheetah Xtend, Reykjavik, Iceland; Freedom Innovations Catapult FX6, Irvine, CA; Ottobock 1E90 Sprinter, Duderstadt, Germany; figure 1). For each RSP model, subjects were aligned with the manufacturer's recommended stiffness category based on their body mass and a high activity level and with ±1 stiffness categories compared with recommended. Recommended height was set so that affected leg length was 2–8 cm longer than unaffected leg length based on the subject's and prosthetist's preference (table 1). Then, RSP height was changed by ±2 cm. We adjusted the height of the C-shaped RSP model using different pylon lengths and adjusted the height of the J-shaped RSP models using a custom aluminium bracket (figure 1).

After RSP alignment, each subject ran on a force-measuring treadmill (Treadmetrix, Park City, UT, USA) at self-selected speeds to acclimate to different RSP configurations until both the subject and prosthetist were satisfied with the alignment and recommended height of each RSP. Following the accommodation session, subjects completed experimental sessions over at least 5 days. For each session, subjects started a series of constant speed trials on the force-measuring treadmill at 3 m s^−1^. If the trial was successful, speed was incremented by 1 m s^−1^ in each subsequent trial until the subject approached their maximum speed, where smaller speed increments were used. A trial was deemed successful if the subject maintained a forward position on the treadmill for 16 consecutive steps. If the trial was unsuccessful, subjects were given the option to try again or accept that speed as their maximum. Subjects had ad libitum rest between trials.

Subjects ran using 15 RSP model, stiffness and height configurations (table 2). First, subjects ran using each RSP model at the recommended and ±1 stiffness categories at recommended height in a randomized order. Then, subjects repeated the trials with the stiffness category for each RSP model that elicited the maximum speed at heights of ±2 cm [2]. These trials were randomly inserted into the trial order.

**Table 2.** Number of subjects for each RSP configuration.

	Freedom Innovations Catapult			Ottobock 1E90 Sprinter			Össur Cheetah Xtend		
	−1 Cat	Rec Cat	+1 Cat	−1 Cat	Rec Cat	+1 Cat	−1 Cat	Rec Cat	+1 Cat
−2 cm	5^d,g,h^	2	2	2	5	2	2	3^i^	5^b,f^
Rec Ht	10^a^	10^a^	10^b^	10	10^e^	10	10^a^	10^a^	10^b^
+2 cm	5^a,c^	2	2	2^c^	5^k^	2^j^	2	3	4^a^

^a^1 subject only completed 3–6 m s^−1^ trials.

^b^2 subjects only completed 3–6 m s^−1^ trials.

^c^1 subject only completed 3–5 m s^−1^ trials.

^d^1 subject only completed 3–6 m s^−1^ trials and force data from 3 and 4 m s^−1^ trials saturated.

^e^1 subject's force data for 3 m s^−1^ trial saturated.

^f^1 subject's force data from 7 m s^−1^ trial saturated.

^g^1 subject lowered RSP height 1.8 cm.

^h^1 subject lowered RSP height 1.3 cm.

^i^1 subject lowered RSP height 1 cm.

^j^1 subject raised RSP height 1 cm.

^k^1 subject raised RSP height 0.8 cm

Rec: recommended; Cat: category; Ht: height.

### 2.3. Data collection

We measured vertical GRFs at 1000 Hz throughout each trial and filtered them with a fourth-order, low-pass Butterworth filter with a 30 Hz cut-off. Reflective markers were placed on the distal end of the RSP and the foot. Three-dimensional marker positions were measured at 200 Hz (Vicon Nexus, Oxford, UK) and filtered with a fourth-order, low-pass Butterworth filter with a 6 Hz cut-off. The RSP and foot markers were used to determine the leg (affected or unaffected) that was in contact with the treadmill.

### 2.4. Data analysis

We corrected for potential force transducer drift using a MATLAB script (Mathworks Inc., Natick, MA, USA) described by Alcantara [21]. Then, we used a custom MATLAB script to calculate *L_c_*, *F*_avg_, and *f*_step_ and the respective symmetry indices. A 20 N vertical GRF threshold was used to determine the start and end of ground contact. As stated previously, average speed (v) equals the product of step length (*L*_step_), and step frequency (*f*_step_)

2.1 $$v=\;L_{\mathrm{step}}\cdot \;f_{\mathrm{step}},$$

where a step includes the stance phase (one foot on the ground) followed by an aerial phase (when neither foot is on the ground) [9]. Therefore, *f*_step_ is equal to

2.2 $$f_{\mathrm{step}}=\;\frac{1}{t_{c}+t_{a}},$$

where *t_c_* is equal to contact time and *t_a_* is equal to aerial time. Furthermore, *L*_step_ depends on contact length (*L_c_*) and the stance average vertical GRF normalized to bodyweight (*F*_avg_) because increasing *F*_avg_ increases the distance travelled during the aerial phase [3]:

2.3 $$\;L_{\mathrm{step}}=L_{c}\;\cdot F_{\mathrm{avg}}.$$

Substituting equation (2.3) into equation (2.1) yields the equation for running speed:

2.4 $$v=L_{c}\;\cdot \;F_{\mathrm{avg}}\;\cdot \;f_{\mathrm{step}}.$$

We calculated the symmetry index (SI) between the affected and unaffected legs using the formula defined by Robinson *et al*. [22] where ‘X’ refers to a biomechanical parameter, 0% indicates perfect symmetry, a positive value indicates a greater value for the unaffected leg than the affected leg, and a negative value indicates a greater value for the affected leg than the unaffected leg:

2.5 $$\;\mathrm{SI}=\;\frac{X_{UL}-\;X_{AL}}{0.5(X_{UL}+\;X_{AL})}\times \;100\text{\%}.$$

### 2.5. Statistical analysis

We constructed linear mixed effects models [23] to test for the effect of RSP model, stiffness category, height and velocity on affected leg *L_c_*, *F*_avg_, *f*_step_ and their respective symmetry indices. The fixed effects in each linear mixed model were RSP model (categorical; Catapult, Sprinter and Xtend), RSP stiffness category (categorical; recommended and ±1 stiffness categories), RSP height (numerical; height relative to recommended height in cm), speed (numerical; speed in m s^−1^) and interactions between each fixed effect and speed. For each comparison, we controlled for the remaining fixed effects. We chose to include interactions with speed because the objective of the study was to not only determine the effect of RSP model, stiffness category, and height on *L_c_*, *F*_avg_, *f*_step_ and their respective symmetry indices, but also to determine how these effects change across speeds. We set the subject as a random effect. We report unstandardized model coefficients (*B*) for each significant association (dependent variable = B*independent variable + intercept). *B* represents the change in the dependent variable related to a unit change in the independent variable. A unit change in SI is a percentage point (p.p.) where one p.p. refers to a 1% unit, such that an increase from 5% to 6% is a 1 p.p. increase as opposed to a 20% increase (i.e. *not* 6%–5%/5% × 100% = 20%). We used a significance level of *p* < 0.05. All statistical tests were done in RStudio (Boston, MA, USA) and packages [23–28].

## 3. Results

We analysed biomechanical variables at 3, 4, 5, 6 and 7 m s^−1^ as each subject achieved a maximum speed of 7 m s^−1^ or faster using most RSP configurations (table 2). Overall, we analysed 704 trials from 10 subjects using different RSP model, stiffness and height configurations at these speeds (table 2). For four subjects, we could not increase or decrease RSP height by 2 cm for one or two RSP models due to the length of each subject's residual limb and height of the RSP components, so we increased or decreased RSP height by 0.8–1.8 cm (table 2). Our statistical models accounted for these heights.

For all RSP configurations, we found that affected leg *L_c_*, *F*_avg_ and *f*_step_ increased with speed (*p* < 2 × 10^−16^; table 3). Average affected leg *L_c_* was 0.67 m and 0.95 m, *F*_avg_ was 1.49 BW and 1.82 BW, and *f*_step_ was 2.91 Hz and 3.97 Hz at 3 m s^−1^ and 7 m s^−1^, respectively. For all RSP configurations, there was no difference in *L_c_* SI or *F*_avg_ SI with faster speed (*p* = 0.55; *p* = 0.90; table 4); however, *f*_step_ SI increased with speed (*p* = 0.013; table 4). The average *f*_step_ SI was 1.90% and 5.21% at 3 m s^−1^ and 7 m s^−1^, respectively, where *f*_step_ was greater in the unaffected versus affected leg.

**Table 3.** Linear mixed model parameters for fixed effects of RSP model, stiffness, height, speed and speed interactions on affected leg (AL) contact length (*L_c_*), stance average vertical ground reaction force (*F*_avg_) normalized to body weight (BW) and step frequency (*f*_step_). Coefficient estimates, 95% confidence intervals for coefficient estimates (*CI*), coefficient standard errors (s.e.), t-values (*t*) and *p*-values (*p*) are listed for each RSP model (Xtend, Sprinter and Catapult); the model coefficients are in reference to the Catapult RSP. There are three RSP stiffness categories: one category (Cat) less stiff (-1) than recommended (Rec), Rec, and one category stiffer than recommended (+1); the stiffness coefficients are in reference to the −1 stiffness category. *P*-values that are significant (*p* < 0.05) are italicized.

	estimate (B)	CI	s.e.	*t*	*p*
AL *L_c_* (m)					
intercept	0.53	[0.49, 0.57]	0.022	24.36	*9.99 × 10^−15^*
model [Sprinter]	0.010	[−0.015, 0.036]	0.013	0.79	0.43
model [Xtend]	−0.037	[−0.062, −0.011]	0.013	−2.78	*0.0056*
stiffness cat [Rec]	−0.034	[−0.059, −0.0088]	0.013	−2.63	*0.0088*
stiffness cat [+1]	−0.064	[−0.090, −0.038]	0.013	−4.85	*1.55 × 10^−6^*
height [cm]	0.0040	[−0.0047, 0.013]	0.0045	0.89	*0.37*
speed [m s^−1^]	0.066	[0.062, 0.070]	0.0022	29.43	*<2 × 10^−16^*
model [Sprinter]*speed [m s^−1^]	0.00036	[−0.0046, 0.0053]	0.0025	0.14	0.89
model [Xtend]*speed [m s^−1^]	0.0016	[−0.0034, 0.0066]	0.0026	0.62	0.54
stiffness cat [Rec]*speed [m s^−1^]	0.0022	[−0.0027, 0.0071]	0.0025	0.88	0.38
stiffness cat [+1]*speed [m s^−1^]	0.0039	[−0.0011, 0.0090]	0.0026	1.53	0.13
height [cm]*speed [m s^−1^]	0.00035	[−0.0014, 0.0020]	0.00087	0.40	0.69
AL *F*_avg_ (BW)					
intercept	1.17	[1.06, 1.29]	0.058	20.28	*5.83 × 10^−14^*
model [Sprinter]	0.14	[0.066, 0.21]	0.036	3.78	*0.00017*
model [Xtend]	0.12	[0.050, 0.19]	0.036	3.34	*0.00089*
stiffness cat [Rec]	0.0035	[−0.066, 0.073]	0.036	0.10	0.92
stiffness cat [+1]	0.076	[0.0051, 0.15]	0.037	2.09	*0.037*
height [cm]	−0.022	[−0.046, 0.0022]	0.012	−1.77	0.077
speed [m s^−1^]	0.078	[0.066, 0.090]	0.062	12.56	*<2 × 10^−16^*
model [Sprinter]*speed [m s^−1^]	0.00050	[−0.013, 0.014]	0.0070	0.072	0.94
model [Xtend]*speed [m s^−1^]	0.0095	[−0.0042, 0.023]	0.0071	1.35	0.18
stiffness cat [Rec]*speed [m s^−1^]	0.0025	[−0.011, 0.016]	0.0069	0.37	0.72
stiffness cat [+1]*speed [m s^−1^]	−0.0057	[−0.020, 0.0082]	0.0071	−0.80	0.43
height [cm]*speed [m s^−1^]	−0.0023	[−0.0070, 0.0024]	0.0024	−0.97	0.33
AL *f*_step_ (Hz)					
intercept	2.12	[1.97, 2.27]	0.079	26.84	*<2 ×10^−16^*
model [Sprinter]	−0.23	[−0.35, −0.11]	0.061	−3.75	*0.00019*
model [Xtend]	−0.13	[−0.25, −0.015]	0.061	−2.19	*0.029*
stiffness cat [Rec]	0.093	[−0.025, 0.21]	0.060	1.54	0.12
stiffness cat [+1]	0.12	[0.00021, 0.24]	0.062	1.95	0.052
height [cm]	−0.015	[−0.056, 0.026]	0.021	−0.72	0.47
speed [m s^−1^]	0.27	[0.25, 0.29]	0.010	26.14	*<2 × 10^−16^*
model [Sprinter]*speed [m s^−1^]	0.022	[−0.00070, 0.045]	0.012	1.89	0.060
model [Xtend]*speed [m s^−1^]	0.0025	[−0.021, 0.026]	0.012	0.21	0.83
stiffness cat [Rec]*speed [m s^−1^]	−0.024	[−0.047, −0.0014]	0.012	−2.07	*0.039*
stiffness cat [+1]*speed [m s^−1^]	−0.026	[−0.050, −0.0030]	0.012	−2.20	*0.029*
height [cm]*speed [m s^−1^]	−0.0061	[−0.014, 0.0018]	0.0041	−1.50	0.13

**Table 4.** Linear mixed model parameters for fixed effects of RSP model, stiffness, height, speed and speed interactions on contact length (*L_c_*), stance average vertical ground reaction force (*F*_avg_) and step frequency (*f*_step_) SI. Coefficient estimates, 95% confidence intervals for coefficient estimates (*CI*), coefficient standard errors (s.e.), *t*–values (*t*) and *p*-values (*p*) are listed for each RSP model (Xtend, Sprinter and Catapult); the model coefficients are in reference to the Catapult RSP. There are three RSP stiffness categories: one category (Cat) less stiff (-1) than recommended (Rec), Rec, and one category stiffer than recommended (+1); the stiffness coefficients are in reference to the −1 stiffness category. *P*-values that are significant (*p* < 0.05) are italicized.

	estimate (*B*)	CI	s.e.	*t*	*p*
*L_c_* SI (%)					
intercept	−6.08	[−9.50, −2.66]	1.75	−3.48	*0.0016*
model [Sprinter]	−0.24	[−2.82, 2.34]	1.32	−0.18	0.86
model [Xtend]	6.90	[4.31, 9.48]	1.33	5.20	*2.69 × 10^−7^*
stiffness cat [Rec]	4.72	[2.17, 7.27]	1.31	3.61	*0.00033*
stiffness cat [+1]	6.40	[3.79, 9.01]	1.34	4.78	*2.17 × 10^−6^*
height [cm]	−2.07	[−2.95, −1.19]	0.45	−4.57	*5.86 × 10^−6^*
speed [m s^−1^]	0.14	[−0.31, 0.58]	0.23	0.60	0.55
model [Sprinter]*speed [m s^−1^]	−0.29	[−0.79, 0.21]	0.26	−1.12	0.26
model [Xtend]*speed [m s^−1^]	−0.81	[−1.31, −0.30]	0.26	−3.11	*0.0020*
stiffness cat [Rec]*speed [m s^−1^]	−0.46	[−0.95, 0.035]	0.25	−1.81	0.071
stiffness cat [+1]*speed [m s^−1^]	−0.43	[−0.94, 0.074]	0.26	−1.67	0.096
height [cm]*speed [m s^−1^]	0.13	[−0.045, 0.30]	0.088	1.43	0.15
*F*_avg_ SI (%)					
intercept	15.03	[9.78, 20.28]	2.71	5.55	*4.33 × 10^−07^*
model [Sprinter]	−10.07	[−14.97, −5.17]	2.52	−4.00	*7.00 × 10^−05^*
model [Xtend]	−5.42	[−10.33, −0.51]	2.52	−2.15	*0.032*
stiffness cat [Rec]	0.77	[−4.07, 5.61]	2.49	0.31	0.76
stiffness cat [+1]	−1.18	[−6.14, 3.78]	2.55	−0.46	0.64
height [cm]	3.86	[2.18, 5.53]	0.86	4.48	*8.58 × 10^−06^*
speed [m s^−1^]	−0.054	[−0.89, 0.78]	0.43	−0.13	0.90
model [Sprinter]*speed [m s^−1^]	−0.061	[−1.01, 0.89]	0.49	−0.13	0.90
model [Xtend]*speed [m s^−1^]	−0.94	[−1.90, 0.020]	0.49	−1.91	0.057
stiffness cat [Rec]*speed [m s^−1^]	−0.053	[−0.99, 0.88]	0.48	−0.11	0.91
stiffness cat [+1]*speed [m s^−1^]	0.24	[−0.72, 1.21]	0.49	0.49	0.62
height [cm]*speed [m s^−1^]	0.088	[−0.24, 0.41]	0.17	0.53	0.60
*f*_step_ SI (%)					
intercept	−2.32	[−5.60, 0.97]	1.70	−1.37	0.17
model [Sprinter]	5.23	[1.78, 8.69]	1.78	2.94	*0.0034*
model [Xtend]	2.99	[−0.48, 6.46]	1.78	1.68	0.094
stiffness cat [Rec]	−1.03	[−4.45, 2.40]	1.76	−0.58	0.56
stiffness cat [+1]	−2.79	[−6.30, 0.71]	1.80	−1.55	0.12
height [cm]	1.98	[0.80, 3.17]	0.61	3.27	*0.0011*
speed [m s^−1^]	0.75	[0.16, 1.35]	0.30	2.48	*0.013*
model [Sprinter]*speed [m s^−1^]	−0.89	[−1.56, −0.22]	0.34	−2.59	*0.0098*
model [Xtend]*speed [m s^−1^]	−0.25	[−0.93, 0.43]	0.35	−0.72	0.47
stiffness cat [Rec]*speed [m s^−1^]	0.57	[−0.092, 1.23]	0.34	1.68	0.094
stiffness cat [+1]*speed [m s^−1^]	0.89	[0.21, 1.57]	0.35	2.54	*0.011*
height [cm]*speed [m s^−1^]	0.10	[−0.13, 0.33]	0.12	0.87	0.39

### 3.1. Prosthesis model

#### 3.1.1. Affected leg biomechanics

**Figure 2.**
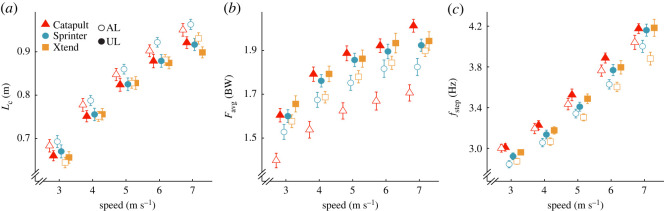
Average ± s.e.m. (*a*) contact length (*L*_c_), (*b*) stance average vertical ground reaction force (*F*_avg_) normalized to body weight (BW) and (*c*) step frequency (*f*_step_) of the affected leg (AL; open shapes) and unaffected leg (UL; solid shapes) for each RSP model across running speeds averaged from three stiffness categories (recommended and ±1) at the recommended RSP height. Colours and shapes refer to the three RSP models (red triangle: Catapult, blue circle: Sprinter, orange square: Xtend). Symbols are offset at each speed for clarity.

In general, use of the Xtend and Sprinter versus Catapult RSPs resulted in shorter or no change in *L_c_*, greater *F*_avg_, and slower *f*_step_ in the affected leg and these differences did not depend on speed. Affected leg *L_c_* was 0.037 m shorter using the Xtend (*p* = 0.0056) compared with the Catapult RSP (table 3; figure 2*a*), and the effect did not depend on speed (*p* = 0.54; table 3; figure 2*a*). We found no differences between affected leg *L_c_* using the Sprinter and Catapult RSPs (*p* = 0.43). Affected leg *F*_avg_ was 0.12 BW greater using the Xtend (*p* = 0.00089) and 0.14 BW greater using the Sprinter (*p* = 0.00017) compared with the Catapult RSP (table 3; figure 2*b*), and the effects did not depend on speed (*p* = 0.54; *p* = 0.89; table 3; figure 2*b*). Affected leg *f*_step_ was 0.13 Hz slower using the Xtend (*p* = 0.029) and 0.23 Hz slower using the Sprinter (*p* = 0.00019) compared with the Catapult RSP (table 3; figure 2*c*) and the effects did not depend on speed (*p* = 0.83, *p* = 0.060; table 3; figure 2*c*).

#### 3.1.2. Asymmetry

**Figure 3.**
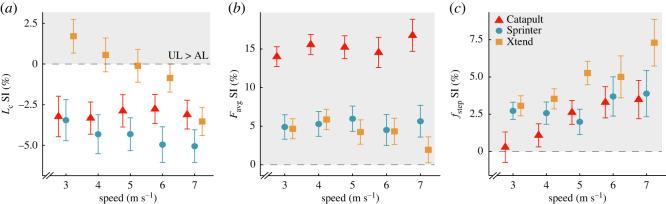
Average ± s.e.m. SI for (*a*) contact length (*L*_c_), (*b*) stance average vertical ground reaction force (*F*_avg_) normalized to body weight and (*c*) step frequency (*f*_step_) for each RSP model across running speeds averaged from three stiffness categories (recommended and ±1) at the recommended RSP height. Colours and shapes refer to the three RSP models (red triangle: Catapult, blue circle: Sprinter, orange square: Xtend). Symbols are offset at each speed for clarity. Horizontal grey dashed line refers to SI = 0 (perfect symmetry). A positive SI (shaded area) indicates a greater unaffected leg (UL) than affected leg (AL) value, and a negative SI indicates a greater AL than UL value.

Overall, the use of the Xtend and Sprinter versus Catapult RSPs decreased or did not change *L_c_* asymmetry, decreased *F*_avg_ asymmetry, and increased or did not change *f*_step_ asymmetry, respectively, and some of these differences depended on speed. We report percentage point (p.p.) changes in SI where one p.p. refers to a 1% unit, such that an increase from 5% to 6% is a 1 p.p. increase. *L_c_* SI was 6.90 p.p. greater (*p* = 2.69 × 10^−07^) using the Xtend compared with the Catapult RSP (table 4; figure 3*a*) and the effect depended on speed so that the difference in *L_c_* SI between the Xtend and Catapult RSPs was attenuated with faster speeds (*p* = 0.0020; table 4; figure 3*a*). *L_c_* SI was negative (longer *L_c_* in the affected than unaffected leg) when using the Catapult RSP across speed, and subjects ran with less asymmetric *L_c_* using the Xtend compared with the Catapult RSP, but this effect was attenuated with faster speed. *F*_avg_ SI was 5.42 p.p. less asymmetric using the Xtend (*p* = 0.032) and 10.07 p.p. less asymmetric using the Sprinter (*p* = 7.00 × 10^−5^) compared with the Catapult RSP (table 4; figure 3*b*) and the effects did not depend on speed (*p* = 0.057, *p* = 0.90; table 4; figure 3*b*). We found no difference in *f*_step_ SI between the Xtend and Catapult RSPs (*p* = 0.094). However, *f*_step_ SI was 5.23 p.p. more asymmetric using the Sprinter compared with the Catapult RSP (*p* = 0.0034; table 4; figure 3*c*) but this effect was attenuated with faster speed (*p* = 0.0098).

### 3.2. Prosthesis stiffness category

#### 3.2.1. Affected leg biomechanics

**Figure 4.**
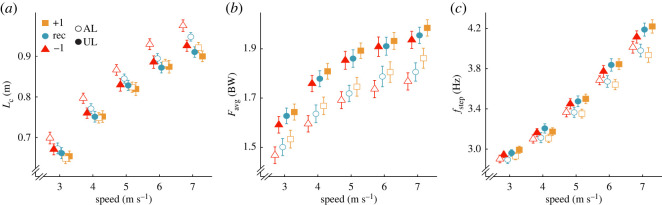
Average ± s.e.m. (*a*) contact length (*L*_c_), (*b*) stance average vertical ground reaction force (*F*_avg_) normalized to body weight (BW) and (*c*) step frequency (f_step_) of the affected leg (AL; open shapes) and unaffected leg (UL; solid shapes) for each RSP stiffness category compared to recommended across speeds averaged across three RSP models (Catapult, Sprinter, and Xtend) at the recommended RSP height. Colours and shapes refer to the three RSP stiffness categories (orange square: +1 category, blue circle: recommended category, red triangle: −1 category). Symbols are offset at each speed for clarity.

In general, use of stiffer compared with less stiff RSP categories decreased *L_c_* and increased *F*_avg_ in the affected leg and these differences did not depend on speed. Regardless of stiffness category, subjects increased affected leg *f*_step_ with faster speed; however, the increase in *f*_step_ with speed was attenuated when subjects used stiffer compared with less stiff RSP categories. Compared with using an RSP one category less stiff than recommended (−1), affected leg *L_c_* was 0.034 m and 0.064 m shorter when using the recommended (*p* = 0.0088) and +1 category RSP (*p* = 1.55 × 10^−6^), respectively (table 3; figure 4*a*), and the effects did not depend on speed (*p* = 0.38, *p* = 0.13; table 3; figure 4*a*). There was no difference in affected leg *F*_avg_ between the −1 and recommended category RSPs (*p* = 0.92; table 3; figure 4*b*). However, compared with using the −1 category RSP, affected leg *F*_avg_ was 0.076 BW greater when using the +1 category RSP (*p* = 0.037) and the effect did not depend on speed (*p* = 0.43; table 3; figure 4*b*). There were no differences in affected leg *f*_step_ between the −1 and recommended category RSPs (*p* = 0.12) or between the −1 and +1 category RSPs (*p* = 0.052). However, relative to the −1 category RSP, for every 1 m s^−1^ faster speed, the increase in affected leg *f*_step_ was attenuated using the recommended (*p* = 0.039) and +1 category RSPs (*p* = 0.029; table 3; figure 4*c*).

#### 3.2.2. Asymmetry

**Figure 5.**
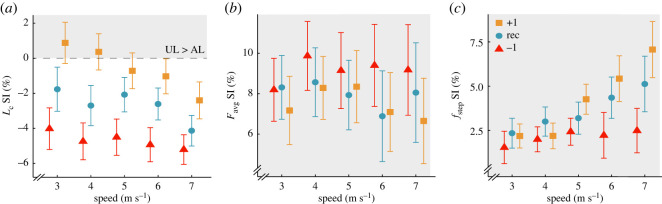
Average ± s.e.m. SI for (*a*) contact length (*L*_c_), (*b*) stance average vertical ground reaction force (*F*_avg_) normalized to body weight and (*c*) step frequency (*f*_step_) for each RSP stiffness category compared to recommended (Rec) across running speeds averaged across three RSP models (Catapult, 1E90 Sprinter, and Xtend) at the recommended RSP height. Colours and shapes refer to the three RSP stiffness categories (orange square: +1 category, blue circle: recommended category, red triangle: −1 category). Symbols are offset at each speed for clarity. Horizontal grey dashed line refers to SI = 0 (perfect symmetry). A positive SI (shaded area) indicates a greater unaffected leg (UL) than affected leg (AL) value, and a negative SI indicates a greater AL than UL value.

Overall, the use of stiffer compared with less stiff RSP categories reduced *L_c_* asymmetry and had no effect on *F*_avg_ asymmetry, and these relationships did not depend on speed. Furthermore, the effect of RSP stiffness category on *f*_step_ asymmetry depended on speed so that when subjects ran at faster speeds, *f*_step_ asymmetry was reduced when using less stiff compared with stiffer RSP categories. Compared with using the −1 category RSP, *L_c_* SI was 4.72 p.p. and 6.40 p.p. higher when using the recommended (*p* = 0.00033) and +1 category (*p* = 2.17 × ^−6^) RSPs, respectively (table 4; figure 5*a*). Since *L_c_* SI was negative for all trials except for the +1 category at 3 and 4 m s^−1^, increasing *L_c_* SI indicates less asymmetry when using a stiffer category RSP. The effects of using the recommended and +1 category RSPs on *L_c_* SI did not depend on speed (*p* = 0.38, *p* = 0.13; table 4; figure 5*a*). There were no differences in *F*_avg_ SI when subjects used the −1 compared with recommended category RSP (*p* = 0.76) or to the +1 category RSP (*p* = 0.64; table 4; figure 5*b*). Moreover, we found no differences in *f*_step_ SI when subjects used the −1 compared with recommended category RSP (*p* = 0.56) or to the +1 category RSP (*p* = 0.12; table 4; figure 5*c*). However, the difference in *f*_step_ SI between the −1 and +1 category RSPs changed with speed (*p* = 0.011) so that subjects ran with similar *f*_step_ asymmetry at slower speeds and less asymmetric *f*_step_ using the −1 compared with +1 category RSP at faster speeds.

### 3.3. Prosthesis height

**Figure 6.**
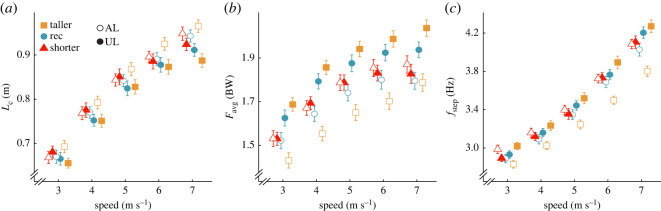
Average ± s.e.m. (*a*) contact length (*L*_c_), (*b*) stance average vertical ground reaction force (*F*_avg_) normalized to body weight (BW) and (*c*) step frequency (*f*_step_) of the affected leg (AL; open shapes) and unaffected leg (UL; solid shapes) for different RSP heights across running speeds averaged across three RSP models (Catapult, Sprinter, and Xtend) with the RSP stiffness category that elicited maximum speed (the category for which RSP height was varied). Colours and shapes refer to the three RSP height groups (orange square: taller, blue circle: recommended, red triangle: shorter). Shorter RSP height refers to −2 cm from the recommended (Rec) height for most of the trials (119/134 trials), but also includes trials where RSP height was decreased by 1.8 cm (5/134 trials), 1.3 cm (5/134 trials) and 1 cm (5/134 trials). Taller RSP height refers to +2 cm from the recommended height for most of the trials (119/129 trials), but also includes trials where RSP height was increased by 1 cm (5/129 trials) and 0.8 cm (5/129 trials). Symbols are offset at each speed for clarity.

**Figure 7.**
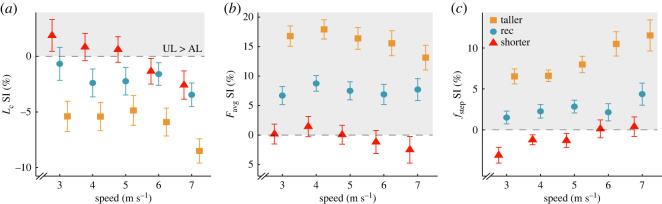
Average ± s.e.m. SI for (*a*) contact length (*L*_c_), (*b*) stance average vertical ground reaction force (*F*_avg_) normalized to body weight and (*c*) step frequency (*f*_step_) for each RSP height across running speeds averaged across three RSP models (Catapult, Sprinter, and Xtend) with the RSP stiffness category that elicited maximum speed (the category for which RSP height was varied). Colours and shapes refer to the three RSP height groups (orange square: taller, blue circle: recommended, red triangle: shorter). Shorter RSP height refers to −2 cm from the recommended (Rec) height for most of the trials (119/134 trials), but it also includes trials where RSP height was decreased by 1.8 cm (5/134 trials), 1.3 cm (5/134 trials) and 1 cm (5/134 trials). Taller RSP height refers to +2 cm from the recommended height for most of the trials (119/129 trials), but it also includes trials where RSP height was increased by 1 cm (5/129 trials) and 0.8 cm (5/129 trials). Symbols are offset at each speed for clarity. Horizontal grey dashed line refers to SI = 0 (perfect symmetry). A positive SI (shaded area) indicates a greater unaffected leg (UL) than affected leg (AL) value, and a negative SI indicates a greater AL than UL value.

Overall, RSP height did not influence affected leg *L_c_*, *F*_avg_ and *f*_step_, but increasing RSP height increased *L_c_*, *F*_avg_ and *f*_step_ asymmetry, and these effects did not depend on speed. We found no significant effect of RSP height on affected leg *L_c_*, *F*_avg_ and *f*_step_ (*p* = 0.37, *p* = 0.077, *p* = 0.47; table 3; figure 6). For every 2 cm increase in RSP height, *L_c_* SI decreased by 4.14 p.p. (*p* = 5.86 × 10^−6^; table 4; figure 7*a*). Since *L_c_* SI was negative for all trials except for the −2 cm RSP height at 3, 4, and 5 m s^−1^, decreasing *L_c_* SI with increasing RSP height indicates greater *L_c_* asymmetry, and this effect did not depend on speed (*p* = 0.15; table 4; figure 7*a*). For every 2 cm increase in RSP height, *F*_avg_ SI increased by 7.72 p.p. (*p* = 8.58 × 10^−6^; table 4; figure 7*b*), which indicates greater *F*_avg_ asymmetry. This effect of RSP height on *F*_avg_ SI did not depend on speed (*p* = 0.60; table 3; figure 7*b*). For every 2 cm increase in RSP height, *f*_step_ SI increased by 3.96 p.p. (*p* = 0.0011; table 4; figure 7*c*) which indicates greater *f*_step_ asymmetry. The effect of RSP height on *f*_step_ SI did not depend on speed (*p* = 0.39; table 4; figure 7*c*).

## 4. Discussion

### 4.1. Prosthesis model

From 3–7 m s^−1^, the use of the Xtend RSP resulted in shorter affected leg *L_c_* compared with the other RSP models; the use of the Sprinter and Xtend RSPs increased affected leg *F*_avg_ and decreased *f*_step_ compared with the Catapult RSP, and these relationships between RSP models were independent of speed, which supported our hypothesis (1a). The results for affected leg *L_c_*, *F*_avg_ and *f*_step_ from 3 to 7 m s^−1^ are similar to overall *L_c_*, *F*_avg_ and *f*_step_ of previous studies at 2.5–3.0 m s^−1^ and maximum speed when athletes with unilateral TTA used different RSP models [1,2] and confirm that the relationships between RSP model and affected leg biomechanics are consistent across a range of speeds.

The use of the J-shaped Sprinter and Xtend RSPs had different effects on affected leg *L_c_* compared with the C-shaped Catapult RSP. This may be related to differences in stiffness values between the recommended categories, where the Xtend is 20–28% and 3–21% stiffer than the Sprinter RSP at 3 and 6 m s^−1^, respectively [8]. To determine if stiffness in kN m^−1^ accounted for differences between the Sprinter and Xtend RSPs, we completed a *post hoc* analysis where we estimated RSP stiffness in kN m^−1^ based on Beck *et al*. [8] and constructed a linear mixed effects model to test for the effect of RSP model, stiffness in kN m^−1^, height and speed on *L_c_*, *F*_avg_ and *f*_step_ (Appendix). We found that when controlling for stiffness in kN m^−1^, there was an interaction between RSP model and running speed so that at 3 m s^−1^, the use of the Sprinter RSP did not change affected leg *L_c_* and the use of the Xtend RSP decreased *L_c_* compared with the Catapult RSP, but at 7 m s^−1^, the use of the Sprinter and Xtend RSPs decreased affected leg *L_c_* compared with the Catapult RSP (*p* = 6.27 × 10^−8^; *p* = 2.84 × 10^−9^; Appendix). Therefore, when controlling for RSP stiffness in kN m^−1^ the effect of the Sprinter and Xtend RSP models on affected leg *L_c_* is similar. Moreover, J-shaped RSPs have lower hysteresis and therefore better energy return compared with the C-shaped RSP [8], which could affect vertical GRF during the second half of stance and may partially explain the increase in affected leg *F*_avg_ when using the J-shaped compared with C-shaped RSPs. Also, because J-shaped RSPs are up to 2.5 cm wider than the C-shaped RSP [1,2], this additional base of support could result in reduced mediolateral foot placement variability [10] and improved dynamic stability, which can be estimated by the maximal Lyapunov exponent, and/or balance, which can be estimated by whole-body angular momentum [29,30]. Previous studies have shown that increased stability during walking led to lower *f*_step_ [31]. Thus, the use of a J-shaped versus C-shaped RSP may increase stability and lower *f*_step_. The use of different RSP models changed affected leg *L_c_*, *F*_avg_ and *f*_step_, which impacted athletes' biomechanical asymmetry across running speeds.

The use of the Xtend compared with Catapult RSP resulted in less asymmetric *L_c_*, and this relationship was attenuated with faster speed, which partially supported our hypothesis (1b). We found that use of the Sprinter and Xtend compared with the Catapult RSP resulted in less asymmetric *F*_avg_, and this relationship did not depend on speed, which supported our hypothesis. We found that the use of the Sprinter compared with the Catapult RSP resulted in more asymmetric *f*_step_, and this relationship was attenuated with faster speed, which contradicted our hypothesis.

Although the effect of RSP model on affected leg *L_c_* was independent of speed, the effect of RSP model on *L_c_* asymmetry depended on speed. *L_c_* SI was near constant across speed for the Sprinter and Catapult RSPs, but *L_c_* SI for the Xtend RSP increased from a negative (longer *L_c_* in the affected than unaffected leg) to a positive value across speeds so that *L_c_* asymmetry was minimized at 5 m s^−1^. This trend may be because the recommended stiffness category of the Xtend is stiffer than that of the Sprinter RSP [8]. In fact, the trend in *L_c_* asymmetry across speeds for the +1 stiffness category for all RSP models is similar to the trend for the recommended category of the Xtend RSP. Furthermore, subjects ran with less asymmetric *F*_avg_ using the J-shaped Sprinter and Xtend RSPs compared with the C-shaped Catapult RSP across speeds, which may be due to height or sagittal plane alignment differences between the RSP models [1]. Although the effect of RSP model on affected leg *f*_step_ was independent of speed, the effect of RSP model on *f*_step_ asymmetry depended on speed. *f*_step_ SI increased at faster speeds using the Xtend and Catapult RSPs, but *f*_step_ SI was nearly constant across speeds using the Sprinter RSP.

Overall, the relationship between RSP model and biomechanical asymmetry depended on speed for *L_c_* and *f*_step_, but not *F*_avg_. The effect of RSP model on *L_c_* and *f*_step_ asymmetry depended on speed even though the effect of affected leg *L_c_* and *f*_step_ did not depend on speed, meaning there may be a speed-dependent effect of RSP model on unaffected leg *L_c_* and *f*_step_. Therefore, a prosthetist may want to consider the intended running speed when prescribing an RSP model that reduces biomechanical asymmetry, which could reduce injury risk. For example, a prosthetist could prescribe the Xtend RSP to minimize *L_c_* asymmetry at 3–5 m s^−1^ or a Sprinter RSP to minimize *f*_step_ asymmetry at 5–7 m s^−1^. Nevertheless, the speed-independent increase in affected leg *F*_avg_ with use of the tested J-shaped compared with C-shaped RSPs allowed subjects to run with less asymmetric *F*_avg_ at all speeds. Athletes may benefit from using the J-shaped RSPs that we tested when running at different speeds because this could reduce *F*_avg_ asymmetry and presumably unaffected leg osteoarthritis risk [20]. Moreover, future studies are warranted to better understand how the alignment and geometry of an RSP model affects biomechanical asymmetry across speeds, which could inform RSP design.

### 4.2. Prosthesis stiffness category

The use of a stiffer compared with less stiff RSP category decreased affected leg *L_c_* and increased affected leg *F*_avg_, which supported our hypothesis (2a) and was similar to overall *L_c_* and *F*_avg_ from running at 2.5 and 3 m s^−1^ when athletes with unilateral TTA used different stiffness category RSPs [1]. However, we found that the effect of RSP stiffness category on affected leg *L_c_* and *F*_avg_ was independent of speed, which contradicted our hypothesis. Furthermore, the effect of RSP stiffness category on affected leg *f*_step_ depended on speed, so that the increase in *f*_step_ with speed was attenuated with a stiffer versus less stiff RSP category, which contradicted our hypothesis. The use of different RSP stiffness categories changed affected leg *L_c_*, *F*_avg_, and *f*_step_ which impacted athletes’ biomechanical asymmetry across running speeds.

The use of a stiffer compared with less stiff RSP category decreased *L_c_* asymmetry, which supported our hypothesis (2b). However, the relationship between RSP category and *L_c_* asymmetry did not depend on speed, and RSP stiffness category did not affect *F*_avg_ or *f*_step_ asymmetry, which contradicted our hypothesis. Subjects decreased *L_c_* asymmetry with increased RSP stiffness category by decreasing their affected leg *L_c_* and keeping their unaffected leg *L_c_* near constant. The increase in affected leg *F*_avg_ with increased RSP stiffness category did not reduce asymmetry, instead the increase in *F*_avg_ corresponded with a similar increase in unaffected leg *F*_avg_. Perhaps, athletes could train with stiffer RSPs and learn to use the increase in affected leg *F*_avg_ and decrease their unaffected leg *F*_avg_ to run with less asymmetric *F*_avg_. The increase in affected leg *f*_step_ with speed was attenuated for the stiffer compared with less stiff RSP categories. Meanwhile, the effect of RSP stiffness category on unaffected leg *f*_step_ had the opposite trend with speed so that the use of a stiffer versus less stiff RSP category increased *f*_step_ asymmetry at faster speeds.

Overall, the relationship between RSP stiffness category and biomechanical asymmetry depended on speed for *f*_step_, but not *L_c_* or *F*_avg_. To decrease *L_c_* asymmetry, prosthetists should prescribe RSPs that are stiffer than recommended; however, there is a trade-off at faster speeds such that increasing RSP stiffness to decrease *L_c_* asymmetry would lead to an increase in *f*_step_ asymmetry. Novel RSP designs that can dynamically adjust their stiffness may be needed to improve biomechanical asymmetry across a range of speeds in athletes with unilateral TTA.

### 4.3. Prosthesis height

The use of an RSP with heights within ±2 cm of recommended did not affect affected leg *L_c_*, *F*_avg_ and *f*_step_, and there was no interaction between the effect of height and speed, which supported our hypothesis (3a). Our results from 3 to 7 m s^−1^ are similar to those from a previous study that found there was no effect of using an RSP with heights within ±2 cm on overall *L_c_* and *F*_avg_ when running at 2.5–3 m s^−1^ [1]. The use of an RSP up to 2 cm taller than recommended height increased *L_c_*, *F*_avg_ and *f*_step_ asymmetry, which supported our hypothesis (3b). Although we did not find significant changes in affected leg biomechanics when using an RSP with different heights, RSP height did affect asymmetry. Overall, the relationship between RSP height and biomechanical asymmetry did not depend on speed for any of the parameters, which suggests that prosthetists may not need to consider running speed when prescribing an RSP height. Furthermore, we found that reducing RSP height by 2 cm decreased biomechanical asymmetry for *L_c_*, *F*_avg_ and *f*_step_ across 3–7 m s^−1^, which suggests that prescribing an RSP height that is shorter than recommended could reduce biomechanical asymmetry for athletes with unilateral TTA running at a range of speeds, which may reduce the risk of injury such as osteoarthritis [19,20]. Since recommended RSP height is set so that the unloaded affected leg length is 2–8 cm longer on average than standing unaffected leg length, perhaps prescribing an RSP height so that the affected leg length is similar to the unaffected leg length would decrease biomechanical asymmetry. Future studies are warranted to examine the effects of RSP heights shorter than those examined in this study to determine if there is an RSP height that minimizes asymmetry in athletes with unilateral TTA.

### 4.4. Limitations and conclusion

Our study had some potential limitations. Our conclusions were limited to the RSP models, stiffness categories and heights that were tested. Moreover, the accommodation period given to each athlete may not have been long enough for them to adapt to each RSP configuration. However, we pseudo-randomized the trial order to mitigate any potential training or adaptation effects. Furthermore, the stiffness of the Sprinter and Xtend RSPs depends on RSP height [8], so there may be a confounding effect between RSP height and stiffness.

Ultimately, understanding how the use of different RSP configurations affect biomechanical variables and biomechanical asymmetry between legs across a range of speeds can improve RSP prescription and design for athletes with unilateral TTA. An RSP prescription that optimizes performance and minimizes injury should likely be based on the desired running speed of an athlete. Perhaps, semi-active RSPs could be designed to alter RSP properties and accommodate to running speed. Previous studies have recommended an RSP configuration based on performance metrics such as running economy [1] and maximum speed [2]. The present study can be used to inform RSP prescription and design that reduces asymmetry and injury risk across a range of speeds. Based on our results, prosthetists should prescribe a J-shaped RSP model with a shorter than recommended height to reduce *F*_avg_ asymmetry and potentially reduce injury risk for athletes with TTA. Moreover, an RSP that increases stiffness with running speed could minimize *f*_step_ asymmetry across speeds; however, there is a potential trade-off between decreasing *f*_step_ asymmetry and increasing *L_c_* asymmetry. Finally, we encourage prosthetists and manufacturers to use the equations from the linear mixed effects models to predict biomechanics and biomechanical asymmetry when using different RSP configurations at different speeds to aid in prescription and design (Appendix). Future studies should also examine individual variability to inform more personalized prescriptions. Ultimately, these results further our understanding of how RSP configuration affects biomechanics and biomechanical asymmetry and can be used to inform RSP prescription and design for athletes with unilateral TTA.

## Data Availability

Our data and code can be accessed through the electronic supplementary material [32]. Tacca_data.csv is a csv file of the data from the study. Tacca_code.rmd is an RStudio file of the code used for the statistics and to create the figures for the study. Tacca_code.pdf is a PDF version of the code.

## Appendix A

We calculated RSP stiffness in kN m^−1^ using the force–displacement equations described in Beck *et al*. [8]. First, we used the filtered vertical GRF data to determine peak vertical GRF for each step and averaged seven steps for each subject and trial using a custom MATLAB script. Then, we used the force–displacement equations [8] to estimate RSP displacement. Since the force–displacement equations depend on speed and are reported for 3 and 6 m s^−1^ [8], we calculated RSP displacement for 3 and 6 m s^−1^ and interpolated linearly to determine the RSP displacement at different speeds. Moreover, the force–displacement equations depend on RSP height (vertical distance from the base of the RSP to the attachment point) for the J-shaped RSPs [8]. We used the equations for a 31.5 cm RSP height reported in Beck *et al*. [8] as 31.5 cm was the only reported RSP height common to both the Xtend and Sprinter RSPs. Finally, we calculated RSP stiffness by dividing peak vertical GRF by the RSP displacement.

We constructed linear mixed effects models [23] to test for the effect of RSP model, stiffness in kN m^−1^, height and speed on *L_c_*, *F*_avg_, *f*_step_ and their respective symmetry indices (tables 5 and 6). The fixed effects in each linear mixed effects model were RSP model (categorical; Catapult, Sprinter, Xtend), RSP stiffness (numerical; stiffness in kN m^−1^), RSP height (numerical; height relative to recommended height in cm), speed (numerical; speed in m s^−1^) and interactions between each fixed effect and speed (tables 5 and 6). We set the subject as a random effect.

**Table 5.** Linear mixed model parameters for fixed effects of RSP model, stiffness in kN m^−1^, height, speed and speed interactions on affected leg (AL) contact length (*L_c_*), stance average vertical ground reaction force (*F*_avg_) normalized to body weight (BW) and step frequency (*f*_step_). Coefficient estimates, 95% confidence intervals for coefficient estimates (*CI*), coefficient standard errors (s.e.), *t*-values (*t*) and *p*-values (*p*) are listed for each RSP model (Xtend, Sprinter and Catapult); the model coefficients are in reference to the Catapult RSP. *P*-values that are significant (*p* < 0.05) are italicized.

	estimate (*B*)	CI	s.e.	*t*	*p*
AL *L_c_* (m)					
intercept	0.73	[0.65, 0.82]	0.043	17.16	*<2 × 10^−16^*
model [Sprinter]	0.045	[0.019, 0.070]	0.013	3.40	*0.000706*
model [Xtend]	0.032	[0.0069, 0.058]	0.013	2.49	*0.013*
stiffness [kN m^−1^]	−0.011	[−0.014, −0.0088]	0.0013	−8.97	*<2 × 10^−16^*
height [cm]	0.0028	[−0.0053, 0.011]	0.0042	0.67	0.50
speed [m s^−1^]	0.074	[0.062, 0.087]	0.0065	11.42	*<2 × 10^−16^*
model [Sprinter]*speed [m s^−1^]	−0.015	[−0.020, −0.0097]	0.0028	−5.47	*6.27 × 10^−8^*
model [Xtend]*speed [m s^−1^]	−0.016	[−0.021, −0.011]	0.0026	−6.02	*2.84 × 10^−9^*
stiffness [kN m^−1^]*speed [m s^−1^]	0.00047	[0.000058, 0.00089]	0.00021	2.22	*0.026*
height [cm]*speed [m ^−1^s]	0.00017	[−0.0014, 0.0017]	0.00081	0.21	0.84
AL *F*_avg_ (BW)					
intercept	0.39	[0.19, 0.59]	0.10	3.76	*0.00024*
model [Sprinter]	0.097	[0.029, 0.16]	0.035	2.77	*0.0057*
model [Xtend]	−0.030	[−0.098, 0.038]	0.035	−0.87	0.39
stiffness [kN m^–1^]	0.035	[0.029, 0.042]	0.0033	10.53	*<2 × 10^−16^*
height [cm]	−0.016	[−0.038, 0.0055]	0.011	−1.46	0.15
speed [m s^−1^]	0.12	[0.089, 0.16]	0.017	7.09	*3.36 × 10^−12^*
model [Sprinter]*speed [m s^−1^]	0.026	[0.012, 0.040]	0.0073	3.57	*0.00039*
model [Xtend]*speed [m s^−1^]	0.045	[0.031, 0.059]	0.0070	6.43	*2.48 × 10^−10^*
stiffness [kN m^−1^]*speed[m s^−1^]	−0.0031	[−0.0042, −0.0020]	0.00057	−5.41	*8.70 × 10^−8^*
height [cm]*speed [m s^−1^]	−0.0025	[−0.0067, 0.0017]	0.0022	−1.17	0.24
AL *f*_step_ (Hz)					
intercept	2.49	[2.13, 2.85]	0.18	13.59	*<2 × 10^−16^*
model [Sprinter]	−0.24	[−0.37, −0.11]	0.066	−3.58	*0.00037*
model [Xtend]	−0.10	[−0.23, 0.028]	0.066	−1.53	0.13
stiffness [kN m^−1^]	−0.012	[−0.024, 0.00040]	0.0063	−1.88	0.061
height [cm]	−0.018	[−0.059, 0.023]	0.021	−0.85	0.40
speed [m s^−1^]	0.22	[0.15, 0.28]	0.033	6.59	*9.05 × 10^−11^*
model [Sprinter]*speed [m s^−1^]	0.021	[−0.0060, 0.048]	0.014	1.53	0.13
model [Xtend]*speed [m s^−1^]	−0.0042	[−0.030, 0.022]	0.013	−0.32	0.75
stiffness [kN m^−1^]*speed [m s^−1^]	0.0017	[−0.00042, 0.0038]	0.0011	1.56	0.12
height [cm]*speed [m s^−1^]	−0.0057	[−0.014, 0.0022]	0.0041	−1.40	0.16

**Table 6.** Linear mixed model parameters for fixed effects of RSP model, stiffness in kN m^−1^, height, velocity and velocity interactions on contact length (*L_c_*), stance average vertical ground reaction force (*F*_avg_) and step frequency (*f*_step_) SI. Coefficient estimates, 95% confidence intervals for coefficient estimates (*CI*), coefficient standard errors (s.e.), *t*-values (*t*) and *p*-values (*p*) are listed for each RSP model (Xtend, Sprinter and Catapult); the model coefficients are in reference to the Catapult RSP. *P*-values that are significant (*p* < 0.05) are italicized.

	estimate (*B*)	CI	s.e.	*t*	*p*
*L_c_* SI (%)					
intercept	−15.28	[−22.76, −7.78]	3.86	−3.96	*9.15 × 10^−5^*
model [Sprinter]	−4.11	[−6.79, −1.42]	1.38	−2.99	*0**.**0029*
model [Xtend]	0.98	[−1.67, 3.65]	1.36	0.72	0.47
stiffness [kN m^−1^]	0.71	[0.45, 0.96]	0.13	5.40	*9.31 × 10^−8^*
height [cm]	−2.03	[−2.88, −1.18]	0.44	−4.66	*3.84 × 10^−6^*
speed [m s^−1^]	−2.09	[−3.41, −0.76]	0.68	−3.07	*0.0023*
model [Sprinter]*speed [m s^−1^]	1.24	[0.68, 1.80]	0.29	4.31	*1.88 × 10^−5^*
model [Xtend]*speed [m s^−1^]	0.75	[0.20, 1.28]	0.28	2.70	*0.0072*
stiffness [kN m^−1^]*speed [m s^−1^]	0.0080	[−0.036, 0.052]	0.022	0.36	0.72
height [cm]*speed [m s^−1^]	0.15	[−0.011, 0.32]	0.085	1.82	0.070
*F*_avg_ SI (%)					
intercept	17.80	[4.16, 31.34]	6.98	2.55	*0**.**011*
model [Sprinter]	−4.53	[−9.60, 0.51]	2.59	−1.75	0.081
model [Xtend]	1.84	[−3.19, 6.83]	2.57	0.72	0.47
stiffness [kN m^−1^]	−0.38	[−0.86, 0.10]	0.25	−1.56	0.12
height [cm]	3.82	[2.22, 5.42]	0.82	4.65	*4.04 × 10^−6^*
speed [m s^−1^]	4.14	[1.63, 6.64]	1.28	3.23	*0.0013*
model [Sprinter]*speed [m s^−1^]	−2.02	[−3.07, −0.95]	0.54	−3.72	*0.00022*
model [Xtend]*speed [m s^−1^]	−2.73	[−3.74, −1.71]	0.52	−5.26	*1.95 × 10^−07^*
stiffness [kN m^−1^]*speed [m s^−1^]	−0.080	[−0.16, 0.0019]	0.042	−1.90	0.058
height [cm]*speed [m s^−1^]	0.053	[−0.26, 0.37]	0.16	0.33	0.74
*f*_step_ SI (%)					
intercept	−10.66	[−20.51, −0.58]	5.10	−2.09	*0**.**037*
model [Sprinter]	5.13	[1.37, 8.92]	1.94	2.65	*0**.**0083*
model [Xtend]	1.40	[−2.30, 5.17]	1.91	0.73	0.46
stiffness cat [kN m^−1^]	0.31	[−0.049, 0.67]	0.18	1.72	0.085
height [cm]	2.04	[0.84, 3.23]	0.61	3.32	*0.00095*
speed [m s^−1^]	1.61	[−0.26, 3.48]	0.96	1.68	0.094
model [Sprinter]*speed [m s^−1^]	−0.68	[−1.47, 0.10]	0.40	−1.68	0.093
model [Xtend]*speed [m s^−1^]	0.15	[−0.62, 0.89]	0.39	0.38	0.71
stiffness [kN m^−1^]*speed [m s^−1^]	−0.028	[−0.089, 0.034]	0.031	−0.89	0.38
height [cm]*speed [m s^−1^]	0.10	[−0.13, 0.33]	0.12	0.85	0.40

### A.1. Example of linear mixed effects model equations

Since the reported unstandardized model coeffcients (*B*) from the linear mixed effects models represent the change in the dependent variable related to a unit change in the independent variable, the model coefficients can be used to estimate the average dependent variable for a certain RSP configuration and running speed. For example, based on table 3, the average affected leg *F*_avg_ for a given RSP configuration and running speed can be estimated using:

$$\begin{array}{rl} \mathrm{AL}\;F_{\mathrm{avg}}(\mathrm{BW}) & =1.17+0.14\times (\mathrm{Model}\;[\mathrm{Sprinter}])+0.12\times (\mathrm{Model}\;[\mathrm{Xtend}])+0.0035\\ & \;\times (\mathrm{Stiffness}\;\mathrm{Cat}\;[\mathrm{Rec}])+0.076\times (\mathrm{Stiffness}\;\mathrm{Cat}\;[+1])\text{–}0.022\times (\mathrm{Height}\;[\mathrm{cm}])+0.078\\ & \;\times (\mathrm{Speed}\;[m/s])+0.00050\times (\mathrm{Model}\;[\mathrm{Sprinter}])\times (\mathrm{Speed}\;[m/s])+0.0095\\ & \;\times (\mathrm{Model}[\mathrm{Xtend}])\times (\mathrm{Speed}[m/s])+0.0025\times (\mathrm{Stiffness}\;\mathrm{Cat}\;[\mathrm{Rec}])\times (\mathrm{Speed}[m/s])\\ & \text{–}0.0057\times (\mathrm{Stiffness}\;\mathrm{Cat}[+1])\times (\mathrm{Speed}[m/s])\text{–}0.0023\times (\mathrm{Height}[\mathrm{cm}])\times (\mathrm{Speed}[m/s]). \end{array}$$

To estimate affected leg *F*_avg_ when an athlete uses the Catapult RSP model, Model [Sprinter] and Model [Xtend] are set to 0, when they use the Sprinter RSP model, Model [Sprinter] is set to 1 and Model [Xtend] is set to 0, and when they use the Xtend RSP model, Model [Sprinter] is set to 0 and Model [Xtend] is set to 1. To estimate affected leg *F*_avg_ when an athlete uses the -1 stiffness category, Stiffness Cat [Rec] and Stiffness Cat [+1] are set to 0, when they use the recommended stiffness category, Stiffness Cat [Rec] is set to 1 and Stiffness Cat [+1] is set to 0, and when they use the +1 stiffness category, Stiffness Cat [Rec] is set to 0 and Stiffness Cat [+1] is set to 1. Height [cm] is set to the RSP height in cm relative to the recommended RSP height. Speed [m s^−1^] is set to the running speed in m s^−1^. Therefore, the average affected leg *F*_avg_ when using a Sprinter RSP with recommended stiffness category and height at 4 m s^−1^ is equal to 1.64 BW.
